# Multiple splice defects in *ABCA1 *cause low HDL-C in a family with Hypoalphalipoproteinemia and premature coronary disease

**DOI:** 10.1186/1471-2350-10-1

**Published:** 2009-01-08

**Authors:** Jeffrey Rhyne, Myrna M Mantaring, David F Gardner, Michael Miller

**Affiliations:** 1 Department of Medicine, Cardiology Division, University Maryland Medical Center and the Baltimore Veterans Affairs Medical Center, 22 South Greene Street, Baltimore, Maryland, 21201 USA; 2 Department of Medicine, Endocrinology Division, Virginia Commonwealth University Health System, 1250 E Marshall St, Richmond, VA, 23298 USA

## Abstract

**Background:**

Mutations at splice junctions causing exon skipping are uncommon compared to exonic mutations, and two intronic mutations causing an aberrant phenotype have rarely been reported. Despite the high number of functional *ABCA1 *mutations reported to date, splice variants have been reported infrequently. We screened DNA from a 41 year-old male with low HDL-C (12 mg/dL [0.31 mmol/L]) and a family history of premature coronary heart disease (CHD) using polymerase chain reaction single-strand conformation polymorphism (SSCP) analysis.

**Methods:**

Family members with low levels of HDL-C (n = 6) were screened by SSCP for mutations in *ABCA1*. Samples with altered SSCP patterns were sequenced directly using either an ABI 3700 or ABI3730Xl DNA Analyzer. To screen for splicing defects, cDNA was isolated from the proband's RNA and was sequenced as above. A series of minigenes were constructed to determine the contribution of normal and defective alleles.

**Results:**

Two novel splice variants in *ABCA1 *were identified. The first mutation was a single base pair change (T->C) in IVS 7, 6 bps downstream from the exon7/intron7 junction. Amplification of cDNA and allelic subcloning identified skipping of Exon 7 that results in the elimination of 59 amino acids from the first extracellular loop of the ABCA1 protein. The second mutation was a single base pair change (G->C) at IVS 31 -1, at the intron/exon junction of exon 32. This mutation causes skipping of exon 32, resulting in 8 novel amino acids followed by a stop codon and a predicted protein size of 1496 AA, compared to normal (2261 AA). Bioinformatic studies predicted an impact on splicing as confirmed by *in vitro *assays of constitutive splicing.

**Conclusion:**

In addition to carnitine-acylcarnitine translocase (CACT) deficiency and Hermansky-Pudlak syndrome type 3, this represents only the third reported case in which 2 different splice mutations has resulted in an aberrant clinical phenotype.

## Background

Epidemiologic studies have demonstrated an inverse association between high-density lipoprotein cholesterol (HDL-C) and coronary heart disease (CHD) [1].

Although low HDL-C is represented by polygenic influences in the majority of cases [2], up to 10% of the general population may have a monogenic abnormality that produces the low HDL-C phenotype [3]. Of the primary candidate genes regulating HDL metabolism, mutations in *ABCA1 *are the most frequent, accounting for the vast majority of HDL-C deficiency states [3-5]. In the absence of genotypic variation or post-translational modification [6], the encoded 220-kDa glycoprotein mediates efflux of cholesterol and phospholipid from macrophages serving as the initial step of reverse cholesterol transport [7]. However, in the presence of defective *ABCA1*, lipid accumulation may occur in both reticuloendothelial organs and the vasculature [8]. Herein, we report a family with low HDL-C, premature CHD and 2 novel mutations that are both outside *ABCA1 *coding regions.

## Methods

### Study subjects

**Figure 1:**
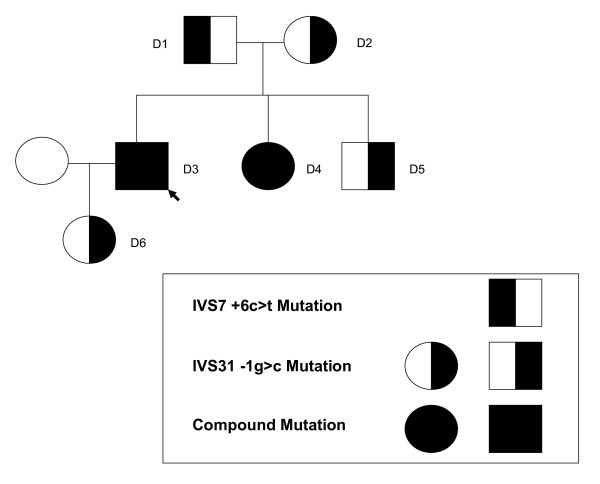
**Pedigree structure of kindred with 2 novel *ABCA1 *splice-site mutations**.

The proband, a 41-year old resident of Virginia, USA, was referred for further evaluation after noted to have reduced HDL cholesterol that varied between 12–18 mg/dL [0.31–0.47 mmol/L]. The patient reported no history of cigarette smoking, hypertension, or diabetes mellitus. Moreover, he has no personal history of symptomatic CHD and is physically active bicycling up to 5 times weekly. There is a family history of premature CHD, characterized by a maternal grandfather who reportedly suffered a myocardial infarction at age 35 years and a maternal brother who underwent percutaneous coronary intervention and stent placement for coronary artery disease at age 56 years. The proband's parents are both living and without symptomatic CHD as are his 2 siblings. A pedigree of the proband and family members participating in the study is shown in Figure 1. On physical examination, the proband did not evidence mucosal discoloration, hepatosplenomegaly or peripheral neuropathy. The protocol was approved by the University of Maryland Institutional Review Board and all subjects gave their informed consent before participation.

### Determination of levels of plasma lipids and lipoproteins

Blood samples were collected after a 12-hour overnight fast. Levels of plasma total cholesterol and triglyceride were measured using enzymatic/colorimetric methods with the Vitros 950 Chemistry Analyzer (Johnson & Johnson, New Brunswick, NJ). HDL-C was determined by the heparin-manganese precipitation method and apolipoproteins A-I and B were measured using a radial immunodiffusion assay [9]. LDL-C was estimated using the Friedewald formula [10].

### Mutation analysis

#### PCR amplification and Single-Strand Conformation Polymorphism (SSCP) analysis

All exons of *ABCA1 *were amplified by PCR from genomic DNA using primers and reaction conditions as previously described [11]. PCR primers amplified all coding regions, splice site junctions, and intronic regions spanning at least 50 nucleotides upstream to the intron-exon junction. Exons of *ABCA1 *were designated using established nomenclature [12]. For mutation analysis of *ABCA1*, PCR products were mixed with 6x loading dye (95% formamide, 20 mmol/L EDTA, 0.05% bromophenol blue, 0.05% xylene cyanol FF), denatured for 10 minutes at 96°C, and placed on ice. SSCP samples were prepared and electrophoresed using nondenaturing 8% or 10% polyacrylamide gels at 5 to 10 watts for approximately 24 hours at room temperature and processed [5].

#### Sequencing of PCR amplified DNA

PCR products showing SSCP shifts of *ABCA1 *were isolated using the Qiagen PCR purification kit and sequenced on an ABI 3700 or ABI3730Xl DNA Analyzer. To rule out changes in other primary HDL candidate genes, all exons and exon-intron boundary regions (including at least 50 bp into the intron region) of the apolipoprotein A-I and lecithin cholesterol acyl transferase (LCAT) genes were amplified and sequenced from proband DNA.

#### Total RNA isolation, cDNA synthesis, and segment amplification

Blood samples were collected into PAXgene Blood RNA tubes (Qiagen, Valencia, CA., USA) from the proband and biologic family members. Total RNA was isolated from the PAXgene tubes using the PAXgene Blood RNA Kit (PreAnalytix/Qiagen, Valencia, Ca., USA). Subsequently, first-strand cDNA synthesis was carried out with the Advantage RT-for-PCR Kit (BD Biosciences, Palo Alto, CA), using oligo dT primers included in the kit.

There were two specific areas of interest in the *ABCA1 *gene that were examined. The region surrounding exon 7 (exon 6 through exon 8) was amplified from cDNA using the following primers: 5'- TGAAGCTTCAAGATTTCCTGG-3' (forward primer in exon 6), and 5'-CTCCTGGGCCAGAGTCCCAAG-3' (reverse primer in exon 8). The region surrounding exon 32 (exon 30 through exon 33) was amplified from cDNA using the following primers: 5'-GCTCCTGAGGACACGGGAACC-3' (forward primer in exon 30), and 5'-CTCATTCACCCAGATCTTGTTC-3' (reverse primer in exon 33) PCR was performed using 20 pmol of each primer in a total volume of 50 μL containing 0.4 mM each dNTP, 100 ng cDNA, 1.0 U of AmpliTaq Gold (Applied Biosystems, Foster City, CA 94404 USA), in 1× Gold Buffer, containing 15 mM Tris-Cl pH8.0, 50 mM KCl and 2.0 mM MgCl^2+ ^(Applied Biosystems). The PCR reaction was carried out in a Techne Genius Thermocycler (Techne Inc, Princeton, NJ), consisting of an initial denaturation step of 95°C for 10 minutes, followed by 35 cycles: denaturation at 94°C for 30 seconds, annealing at 57.5°C for 30 seconds, and extension at 72°C for 45 seconds. A final extension step at 72°C for 5 minutes followed the last PCR cycle. PCR products were separated on a 16% acrylamide gel. The ratios of PCR amplification products were determined by densitometry using the VersaDoc Imaging System (Bio-Rad Laboratories, Hercules, CA.).

#### Cleaning, cloning and screening of *ABCA1 *cDNA fragments

Following PCR, *ABCA1 *cDNA fragments containing exons 6–8, and 30–33 were cleaned for cloning with the Qiaquick PCR Purification Kit (Qiagen, Inc, Valencia, CA.). The PCR products were then cloned into a pDrive Cloning Vector, Qiagen PCR Cloningplus Kit (Qiagen, Inc, Valencia, CA.), plated, and grown overnight. Positive colonies were PCR-screened using the primers listed above. Colonies were grown overnight in 5 mL of LB media at 37°C, with shaking at 220 rpm. Plasmid DNA was isolated using the Quantum Prep Plasmid Miniprep Kit (Bio-Rad Laboratories, Hercules, CA). Sequencing was performed using an ABI 3700 or ABI3730Xl DNA Analyzer.

### Splice site analysis

Both of the base pair changes were analyzed using either the Automated Splice Site Analysis server at  at The University of Western Ontario [13] or the Delila servers [14,15] that also uses information theory methods to identify binding sites on DNA or RNA.

### Constitutive splicing evaluation

#### Primer design and sequences

In addition to the proband and family members, 20 unrelated control samples with various HDL-C levels were used for the constitutive splicing analysis. All of the intron/exon junctions in *ABCA1 *were analyzed using the servers listed above to determine the information content of splice-site junctions. Low acceptor Ri values indicated potential regions of abnormal splicing (Table 1). Primers were then designed to evaluate these regions and determine the extent of constitutive splicing (Table 2).

**Table 1:** Tabular representation of the information content for all acceptor intron/exon junctions of *ABCA1*.

**Intron**	**Acceptor Ri value (bits)**	**Intron**	**Acceptor Ri value (bits)**
1	12.7	26	10.5
2	8.2	27	8.5
3	8.1	28	**1.2**
4	10.7	29	6.1
5	**2.2**	30	14.4
6	5.8	31	**3.4**
7	12.0	32	13.6
8	5.9	33	6.5
9	12.4	34	7.9
10	8.7	35	11.7
11	**3.2**	36	14.0
12	6.0	37	9.1
13	5.8	38	11.7
14	8.5	39	17.1
15	11.9	40	5.3
16	9.6	41	11.9
17	9.7	42	9.6
18	11.7	43	11.4
19	10.9	44	11.5
20	9.2	45	8.6
21	13.0	46	9.5
22	8.2	47	10.2
23	12.2	48	12.7
24	11.2	49	7.3
25	13.7		

The four junctions (**in bold**) were further examined for aberrant splicing events.

**Table 2:** Primers to *ABCA1 *in regions predicting constitutive splicing defects

**Name**	**Sequence**
ABC-A1Ex4FcDNA	5'-GCCATTTTCCAAATAAAGCCATGCC-3'
ABC-A1Ex8RcDNA	5'-CTCCTGGGCGAGAGTCCCAAG-3'
	
ABC-A1Ex10FcDNA	5'-CTCCTTACTGCAATGATTTGATGAA-3'
ABC-A1Ex14RcDNA	5'-CCGCATGTCCTCAAAGGGGTCAGC-3'
	
ABC-A1Ex27FcDNA	5'-ATGGTACCTTGCCAGCAAGACGAA-3'
ABC-A1Ex30RcDNA	5'-GGGATTGGGTTTCCTTCCATACAG-3'
	
ABC-A1Ex30FcDNA	5'-GCTCCTGAGGACACGGGAACC-3'
ABC-A1Ex33RcDNA	5'-CTCATTCACCCAGATCTTGTTC-3'

#### Amplification details

Regions of cDNA were amplified using the primers listed above. PCR cycling conditions included initial denaturation at 95°C for 10 min, followed by 35 cycles of 95°C for 30 s, 55°C for 30 s, and 72°C between 30 s to 1.0 min (depending upon the length of the amplicon) with an additional extension of 72°C for 5 min. Amplifications were performed in a Techne Genius thermocycler (Techne, Burlington, NJ, USA). Samples were amplified using 50 μl reaction volumes consisting of 20 to 100 ng cDNA, 10 pmol each primer, 0.2 mM each dNTP, 1× Gold Buffer, 2.0 mM MgCl2, and 1.25 U AmpliTaq Gold. Amplified cDNA included the proband and lab controls with normal HDL-C.

#### Band excision, purification and re-amplification

Bands corresponding to un-expected amplicon sizes were excised and purified using the QIAEX II Gel Extraction Kit (Qiagen Inc., 27220 Turnberry Lane Suite 200 Valencia, CA 91355). Because of insufficient purified DNA from the bands to permit successful cloning, the DNA was re-amplified using the same original amplification conditions and used directly for cloning.

#### Cloning and colony screening

PCR fragments were cloned into the pJET1 vector (Fermentas Inc., 7520 Connelley Drive, Hanover, MD 21076). Colony screening was performed using the same amplification conditions to test for successful DNA fragment insertion into the pJET1 Cloning Vector.

#### Plasmid mini-prep preparation and sequencing

Positive colonies were grown O/N at 37C. Plasmid minipreps were prepared using the Quantum Prep Plasmid Miniprep Kit (Bio-Rad Laboratories, 2000 Alfred Nobel Drive, Hercules CA, 94547). Plasmids were subjected to direct sequencing in both directions using the primers listed above, as well as, in some cases, primers specific for the pJET vector (pJET1-Forward and pJET1-Reverse). Sequencing reactions were cleaned using Performa V3 96-Well Short Plates (Edge BioSystems, 201 Perry Parkway, Suite 5 Gaithersburg, MD 20877) and run on an ABI 3730XL automated sequencer (Applied Biosystems, Foster City, CA, USA).

#### Construction of ABCA1 minigenes

To investigate the precise contribution to aberrant splicing of each of the two intronic mutations (IVS7 +6t>c and IVS31-1g>c), wild-type and mutant *ABCA1 *minigenes were constructed using the proband's gDNA as a template. The first pair of constructs were designated WT_IVS31-1g>c (wild-type) and MT_IVS31-1g>c (mutant). These two constructs were amplified using forward primer (IVS30) 5'-GGGCATACACCTCCATGAAGGCAG-3' and the reverse primer (IVS34A) 5'-ATGAGCTGCCAGGTGATCCGTTTA-3'. PCR cycling conditions included initial denaturation at 92°C for 5 min, followed by 35 cycles of 95°C for 30 s, 55°C for 30 s, and 72°C for 5.0 min, with an additional extension of 72°C for 10 min. Samples were amplified using 50 μl reaction volumes consisting of 100 ng gDNA, 10 pmol each primer, 0.2 mM each dNTP, 1× Dynazyme EXT buffer (F-514), and 1.0 U Dynazyme EXT(New England Biolabs, Ipswich, MA, USA). This insert was composed of a small portion of the 5' end of intron 30, exon 31, intron 31, exon 32, intron 32, exon 33, intron 33, exon 34, and the 3' portion of intron 34, and is 4.87 kb in size. The two constructs were then cloned into the pTargeT vector (Promega) for minigene expression studies.

The second pair of constructs were designated WT_ IVS7 +6t>c (wild-type) and MT_ IVS7 +6t>c (mutant). Because several intronic regions were too large to be incorporated in the vector (IVS6, 2.98kb; IVS7, 12.96 kb; and IVS8, 4.96 kb) truncated intronic regions of approximately 400 bp were used (**see Primers**, Table 3).

**Table 3:** Primers used for construct amplification of the IVS7 variant

**Segment 1**	**Primer Sequence**
IVS 5-3' *BamHI*	5'-GATGGGGATCCCAGCACCATCACACTGTTTGAG-3'
IVS6-5' *XhoI*	5'-CGGCGCTCGAGGAAATCATCACAAATGCCAAG-3'
	
**Segment 2**	
IVS 6-3'	5'-CGCTCGAGGATGTTGTTCATGTAATAAA-3'
IVS 7-5' *HindIII*	5'-CGAAGCTTCCAACTGTGACAGCCCACAA-3'
	
**Segment 3**	
IVS 7-3' *HindIII*	5'-GCAAGCTTGTGAGGCGTCATTAACTTGT-3'
IVS 8-5' *PvuI*	5'-GCCGATCGTTATTCCTGTTAGTTAGCAA-3'
	
**Segment 4**	
IVS 8-3' *PvuI*	5'-GCCGATCGGCAGAGGGGTCTCAGAATCC-3'
IVS 9-5'	5'-CCTTCTGCCATTATCTCATTCTTG-3'

PCR cycling conditions included initial denaturation at 95°C for 10 min, followed by 35 cycles of 95°C for 30 s, 55°C for 30 s, and 72°C for 45s, with an additional extension of 72°C for 5 min. Amplifications were performed in a Techne Genius thermocycler (Techne, Burlington, NJ, USA). Samples were amplified using 50 μl reaction volumes consisting of 20 to 100 ng cDNA, 10 pmol each primer, 0.2 mM each dNTP, 1× Gold Buffer, 2.0 mM MgCl2, and 1.25 U AmpliTaq Gold. Four segments were generated; however, only the 3 terminal segments ligated correctly, and these were cloned into the pTargeT vector. Segment 1 was redesigned and cloned upstream to the 3 terminal segments. All construct sequences were confirmed by sequencing. The total size of the insert was 3.5 kb in size, which combined with the pTargeT vector (5.69 kb), resulted in a total insert + vector size of 9.19 kb.

### Expression of *ABCA1 *minigenes in transfected cells

COS cells were grown in DMEM containing 5% FBS at 37°C and 5% CO_2 _and used for transfection. On the day prior to transfection, 4 × 10^5 ^cells were seeded on 6 well plates in 3.0 milliliters of media. On the day of transfection, 1.5 μg of the pTargeT plasmids containing the various constructs were transfected using Polyfect Transfection Reagent (Qiagen, Valencia, CA). Forty-eight hours post transfection, total cellular RNA was isolated using the High Pure RNA Isolation Kit (Roche, Mannheim Germany). First-strand cDNA synthesis was carried out with the Advantage RT-for-PCR Kit (BD Biosciences, Palo Alto, Ca), using random decamers included in the kit. cDNA amplification was performed using the appropriate primer pairs (IVS 7, IVS7 For 5'-TGAAGCTTCAAGATTTCCTGG-3' + IVS7 Rev 5'-CTCCTGGGCCAGAGTCCCAAG-3', and for IVS31, IVS31 For 5'-CTGCCAGGCAGGGGAGGAAGAGTG-3' + IVS31 Rev 5'-CTCATTCACCCAGATCTTGTTC-3'). In both cases, a very small amount of endogenous *ABCA1 *was amplified from COS1 cells and was subsequently subtracted from all samples in densitometry analysis.

## Results

Levels of lipids, lipoproteins and apolipoproteins AI and B are shown in Table 4. All family members had reduced HDL-C and apoAI with the lowest levels identified in the proband (12 mg/dL [0.31 mmol/L]) and his sister (22 mg/dL [0.57 mmol/L]). An altered SSCP pattern was identified in two regions of *ABCA1 *encompassing IVS7 and IVS31 in the proband and affected family members with low HDL-C but was not observed in an additional 100 screened unrelated subjects. Direct sequencing of these regions revealed alteration in the splice-site junction suggestive of two novel splice variants in *ABCA1*.

**Table 4:** Selected baseline characteristics including age (years), body mass index (kg/m^2^), lipids, lipoproteins (mg/dL and mmol/L) apolipoproteins (mg/dL) and presence or absence of ABCA1 splice variants of the low HDL pedigree

**Relative**	**Age**	**BMI**	**TC**	**TG**	**HDL**	**LDL**	**ApoAI**	**ApoB**	**IVS7+6c>t**	**IVS31-1g>c**
Father (D1)	65	38.3	189	245	35	105	149	110	Yes	No
***(mmol/L)***			***4.90***	***2.77***	***0.91***	***2.72***				
										
Mother (D2)	63	30.9	155	293	31	65	139	87	No	Yes
***(mmol/L)***			***4.01***	***3.31***	***0.80***	***1.68***				
										
**Proband (D3)**	**41**	**28.2**	**101**	**56**	**12**	**65**	**70**	**59**	**Yes**	**Yes**
***(mmol/L)***			***2.62***	***0.63***	***0.31***	***1.68***				
										
Sister (D4)	37	29.3	149	70	22	113	73	90	Yes	Yes
***(mmol/L)***			***3.86***	***0.79***	***0.57***	***2.93***				
										
Brother (D5)	43	34.6	159	176	24	100	109	94	No	Yes
***(mmol/L)***			***4.12***	***1.99***	***0.62***	***2.59***				
										
Daughter (D6)	16		74	42	35	31	100	25	No	Yes
***(mmol/L)***			***1.92***	***0.47***	***0.91***	***0.80***				

### Splice site analysis

Two servers (Automated Splice Site Analysis server at  University of Western Ontario and the Delila servers) were used to examine both mutation sites. Analysis of the IVS7 +6t>c mutation predicts a decrease in the strength of the splice site, from 10.2 bits to 8.8 bits of information, resulting in a 2.7 fold decrease in binding efficiency and a reduction in binding from 100% for normal vs. 37.4% for the mutant. This creates a "leaky site" that would be predicted to adversely affect normal protein production. Information analysis also predicts the complete loss of splice site recognition for IVS31-1g>c, with original information content of the splice site being reduced from 3.4 bits to -3.9 bits. Both of these predicted results compare favorably with original densitometry measures, assuming a somewhat lower stability for malformed proteins [16,17].

### Ex Vivo cDNA analysis

**Figure 2:**
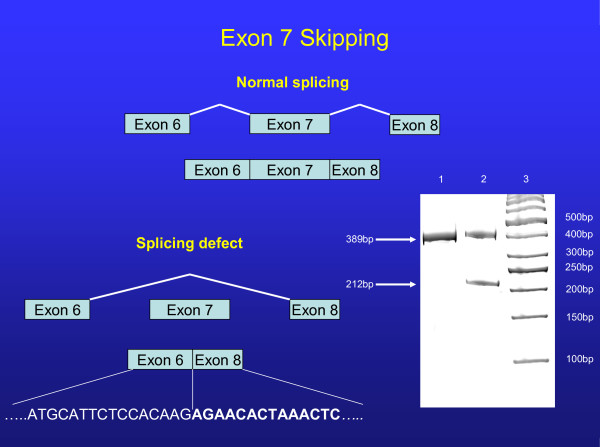
**Normal splicing of exon 7 is illustrated in the top portion of the figure**. Schematic representation of exon 7 skipping is shown at the lower portion. A 16% acrylamide gel run of the PCR products from cDNA shows the normal splice product of 389 base-pairs and a splice variant band of 212 base-pairs that results in the elimination of 59 amino acids. Lane 1 (control), Lane 2 (proband). The referent 50 base-pair ladder is shown in lane 3. Sequence of aberrant splicing shown at bottom of figure.

**Figure 3:**
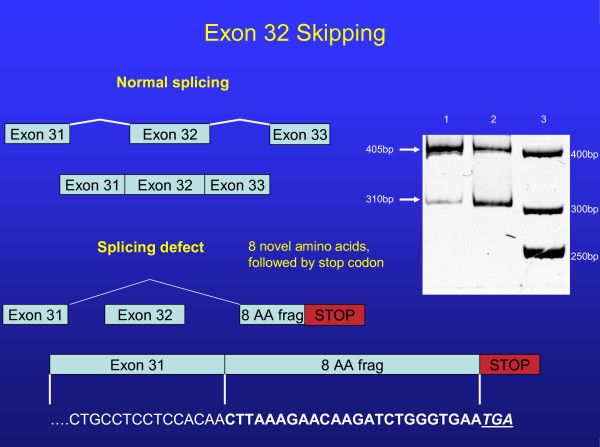
**Normal splicing of exon 32 is illustrated in the top portion of the figure**. Schematic representation of exon 32 skipping is shown at the lower portion. A 16% acrylamide gel run of the PCR products from cDNA shows the normal splice product of 405 base-pairs and a splice variant band of 310 base-pairs that results in the addition of 8 amino acids followed by a stop codon. The predicted protein size is 1496 amino acids compared to normal (2261 amino acids). Lane 1 (control), Lane 2 (proband). Note the constitutive splicing in control lane as well as affected. Referent 50 base-pair ladder is shown in lane 3. Sequence of aberrant splicing shown at bottom of figure.

The first mutation was a single base pair change (T→C) in IVS 7, 6 base-pairs downstream from the exon7/intron7 junction (Nomenclature IVS7+6t>c). Following cDNA amplification, a smaller band corresponding to Exon 7 skipping was observed (Figure 2). Subcloning of alleles confirmed Exon 7 skipping in one allele that resulted in the elimination of 59 Amino Acids in the first extracellular loop of the ABCA1 protein but did not result in a frameshift or premature truncation. The second mutation was a single base pair change (G→C) at IVS 31 -1, at the intron/exon junction of Exon 32 (Nomenclature IVS31-1g>c) (Figure 3). This mutation causes skipping of exon 32, resulting in 8 novel amino acids followed by a stop codon with a predicted protein size of 1496AA, compared to normal (2261AA). In contrast, sequencing of the *APOA1 *and *LCAT *promoter splice and coding regions disclosed no mutations.

### Minigene transfection results

**Figure 4:**
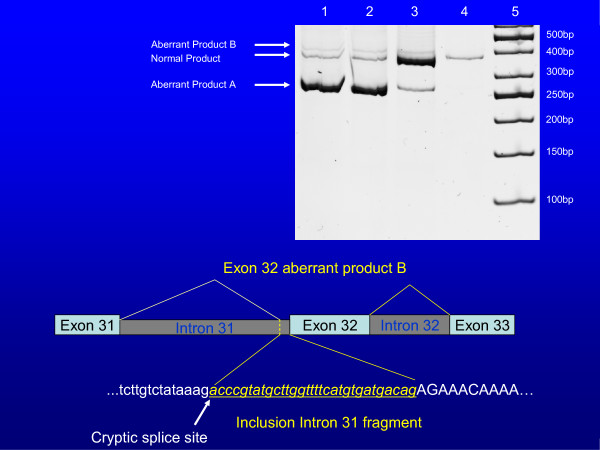
**Top: Post transfection PCR products of the minigene construct for IVS31**. Lanes 1 and 2 = IVS31-1g>c (mutant); Lane 3 = IVS31-1g (wildtype); Lane 4 = untransfected control; Lane 5 = 50 bp ladder. Bottom: Sequence of aberrant product B containing Exon 31,  activation of a cryptic splice site with inclusion of a potion of intron 31 (31 base-pairs), and retention of Exon 32.

Using the minigene construct, the IVS31 splicing defect demonstrated results as predicted from bioinformatic analysis. Specifically, the mutant construct revealed 98% of aberrantly spliced product A by densitometry, with the remaining 2% comprising the aberrantly spliced product B (constitutively produced). The wild-type sample showed 89% of normally spliced product, 9% of aberrantly spliced product A, with the remaining 2% of the other species (B) of aberrantly spliced product (Figure 4) for sequence of included intron 31 base pairs. This demonstrates that the mutation in question (IVS31-1g>c) completely abolishes normal splicing, and confirms the results generated using Automated Splice Site Analysis. This result is consistent with the original observation of constitutive aberrant splicing at a low level. In contrast, the IVS7 mutation did not reveal the level of mis-splicing anticipated from cDNA analysis because only 2–3% of defective product was detected.

## Other potential constitutive splice sites investigated

A total of 4 intron/exon junctions that showed low information content were examined for altered splicing events (Table 1). The intron/exon junction 5/6 revealed normal splicing. In contrast, the 3 other sites examined demonstrated low levels of aberrant splicing.

Specifically, at the intron 11/exon 12 junction, an aberrant protein product is produced. It consists of correct splicing through exon 11, followed by exons 12 and 13 that are spliced out of the final product. This results in the inclusion of 8 novel amino acids after Exon 11 followed by a stop codon. Secondly, at the intron 28/exon 29 junction, a splicing defect resulted in the splicing out of exon 29. This product then contains a novel amino acid followed by a stop codon. Finally, at the intron 31/exon 32 junction, in addition to the previously discovered splicing mutation (exon 32 spliced out), an alternative acceptor site in IVS31 was identified creating 50 novel amino acids, followed by a stop codon. All 3 of these splicing defects were found to occur constitutively at a level less than 1% of the normal transcript.

## Discussion

Following the discovery that mutations in *ABCA1 *cause Tangier Disease or familial hypoalphalipoproteinemia [18-20], more than 90 functional variants [4,8,21] have now been reported to cause the low HDL-C phenotype. The vast majority of these mutations are located in coding regions, whereas only a few involve intronic areas [4,8,21,22]. In the present study, alterations in two different intronic regions of *ABCA1 *were identified resulting in skipping of exons 7 and 32, the former resulting in removal of 59 amino acids from the final protein product and the latter causing a frameshift with a stop codon predicting premature truncation at 1496 AA, compared to the normal protein, 2261AA.

That the IVS7 mutation did not reveal the level of mis-splicing expected in the minigene experiment is unclear. One possibility is the potential influence of non-included intronic regions. That is, while the IVS7+6t>c mutation is necessary for mis-splicing to occur, there may be additional factors required to produce the high levels of mis-splicing predicted from cDNA analysis. For example, the size of the intronic regions surrounding the IVS7 mutation may have been excessively large (13 kb) to permit conditions favoring optimization of the minigene construct. To this end, Ladd and Cooper [23] identified splicing enhancers and suppressors at appreciable distances from exon/intron junctions. Moreover, Genetta *et al *[24] discovered a bipartite intronic splicing enhancer located 2.6 kb downstream from the intron/exon junction required for exon inclusion. Gatto *et al*. [25] investigated intronic regions that control splicing of a fibroblast growth factor receptor-2 alternative exon and identified a region located 1 kb downstream from the exon splice site. This raises the possibility that due to size constraints, intronic enhancers and/or suppressors may have been excluded from our constructs, thereby limiting complete evaluation of these intronic regions.

Although the majority of human mutations are the result of missense or nonsense variation in genomic DNA [26], it has recently been appreciated that aberrant splicing may also account for a significant proportion of cases [27]. Because mutations are proportional to coding length [28], large genes such as *ABCA1*, are more likely to yield splice-mediated functional mutations as demonstrated in the present case. To our knowledge, this represents only the 3^rd ^case of biallelic splicing defects associated with human pathology. [29,30].

In addition to the mutations reported above, we identified 3 additional defects, all constitutive and at very low levels (less than 1% compared to the normal transcript). Two of the newly presented variants result in exon skipping, and the other involves activation of a cyptic splice site. However, because only trace amounts of these variant proteins may be produced, it is highly unlikely that they would bear any physiologic significance. Moreover, the alternate spliced products occurred with equal frequency (as determined by densitometry) in all subjects studied, independent of HDL cholesterol, and therefore unlikely to be under regulatory control [31,32].

Whereas the identification of two intronic mutations in *ABCA1*, located on opposite chromosomes may have predicted the Tangier Disease phenotype, the patient did not exhibit any of the known clinical features of this disorder [33]. This may in part reflect the relative inability of the first splicing defect (IVS7 +6t>c) to completely suppress some normal protein production as confirmed by densitometry and automated splice site analysis. In the present case, the haploinsufficiency was the likely result of the combination of a negative effect of the expressed mutant isoform resulting from protein termination (splice defect at exon 31) and post-translational instability as a byproduct of a poorly or non-functioning protein (splice defect at exon 7) based upon the predicted removal of 59 amino acids within the extracellular loop. In contrast to affected family members with premature CHD, the proband is clinically healthy despite low HDL-C, raising the possibility that in the absence of other CHD risk factors, isolated low HDL-C may not pose a substantially increased risk of vascular disease [34].

The *ABCA1 *splicing mutations observed in the present case resulted in an approximate 30–50% reduction in HDL-C with more modest effects on apo AI levels whereas compound heterozygotes displayed greater reductions in HDL-C (50–80%) and ApoAI as we previously reported [22,35].

To date, we have identified 15 functional mutations in *ABCA1 *(n = 10), *LCAT *(n = 3) *and APOA1 *(n = 2) in 20 unrelated probands with very low HDL-C (20 mg/dL or 0.52 mmol/L); three of these mutations are the result of splicing defects in *ABCA1*. These data suggest that although splice mutations in *ABCA1 *are uncommon, they are worthy of further consideration, particularly in cases where promoter and coding regions of candidate genes fail to identify the genetic basis of very low HDL-C.

## Conclusion

We have identified 2 different intronic variants in *ABCA1*. This represents the first reported case of distinct splice-site mutations causing HDL-C deficiency and the third overall aberrant clinical phenotype occurring as a consequence of multiple splicing defects.

## Competing interests

The authors declare that they have no competing interests.

## Authors' contributions

JR participated in the SSCP screening, performed the cDNA synthesis and sequencing, designed the minigene constructs and experiment and interpreted results, performed the splice site analysis, and helped in the drafting and revision of the manuscript.

MMM performed the SSCP screening.

DG recruited the subject and provided clinical insights into the case.

MM designed the study, and drafted, reviewed and revised the manuscript.

All authors have read and approved the final manuscript.

## Pre-publication history

The pre-publication history for this paper can be accessed here:


